# Healthcare worker stress, anxiety and burnout during the COVID-19 pandemic in Singapore: A 6-month multi-centre prospective study

**DOI:** 10.1371/journal.pone.0258866

**Published:** 2021-10-22

**Authors:** Irene Teo, Junxing Chay, Yin Bun Cheung, Sharon C. Sung, Komal G. Tewani, Li Fang Yeo, Grace Meijuan Yang, Fang Ting Pan, Jin Ying Ng, Fazila Abu Bakar Aloweni, Hui Gek Ang, Tracy Carol Ayre, Crystal Chai-Lim, Robert Chun Chen, Ai Ling Heng, Gayathri Devi Nadarajan, Marcus Eng Hock Ong, Brian See, Chai Rick Soh, Boon Kiat Kenneth Tan, Bien Soo Tan, Kenny Xian Khing Tay, Limin Wijaya, Hiang Khoon Tan

**Affiliations:** 1 Programme in Health Services & Systems Research, Duke-NUS Medical School, Singapore, Singapore; 2 Lien Centre for Palliative Care, Duke-NUS Medical School, Singapore, Singapore; 3 Department of Psychosocial Oncology, National Cancer Centre Singapore, Singapore, Singapore; 4 Centre for Quantitative Medicine, Duke-NUS Medical School, Singapore, Singapore; 5 Centre for Child Health Research, Tampere University, Tampere, Finland; 6 Department of Developmental Psychiatry, Institute of Mental Health, Singapore, Singapore; 7 Department of Gynaecological Oncology, KK Women’s and Children’s Hospital, Singapore, Singapore; 8 Department of Internal Medicine, Changi General Hospital, Singapore, Singapore; 9 Division of Supportive and Palliative Care, National Cancer Centre Singapore, Singapore, Singapore; 10 Division of Nursing, Singapore General Hospital, Singapore, Singapore; 11 Division of Allied Health, Singapore General Hospital, Singapore, Singapore; 12 Medical Social Services, Singapore General Hospital, Singapore, Singapore; 13 Division of Radiological Sciences, Singapore General Hospital, Singapore, Singapore; 14 Department of Emergency Medicine, Singapore General Hospital, Singapore, Singapore; 15 Occupational Health Service, Changi General Hospital, Singapore, Singapore; 16 Department of Anaesthesiology, Singapore General Hospital, Singapore, Singapore; 17 Department of Orthopaedic Surgery, Singapore General Hospital, Singapore, Singapore; 18 Department of Infectious Disease, Singapore General Hospital, Singapore, Singapore; 19 Division of Surgery and Surgical Oncology, Singapore General Hospital and National Cancer Centre Singapore, Singapore, Singapore; 20 SingHealth Duke-NUS Global Health Institute, Singapore, Singapore; China University of Mining and Technology, CHINA

## Abstract

**Aim:**

The long-term stress, anxiety and job burnout experienced by healthcare workers (HCWs) are important to consider as the novel coronavirus disease (COVID-19) pandemic stresses healthcare systems globally. The primary objective was to examine the changes in the proportion of HCWs reporting stress, anxiety, and job burnout over six months during the peak of the pandemic in Singapore. The secondary objective was to examine the extent that objective job characteristics, HCW-perceived job factors, and HCW personal resources were associated with stress, anxiety, and job burnout.

**Method:**

A sample of HCWs (doctors, nurses, allied health professionals, administrative and operations staff; N = 2744) was recruited via invitation to participate in an online survey from four tertiary hospitals. Data were gathered between March-August 2020, which included a 2-month lockdown period. HCWs completed monthly web-based self-reported assessments of stress (Perceived Stress Scale-4), anxiety (Generalized Anxiety Disorder-7), and job burnout (Physician Work Life Scale).

**Results:**

The majority of the sample consisted of female HCWs (81%) and nurses (60%). Using random-intercept logistic regression models, elevated perceived stress, anxiety and job burnout were reported by 33%, 13%, and 24% of the overall sample at baseline respectively. The proportion of HCWs reporting stress and job burnout increased by approximately 1·0% and 1·2% respectively per month. Anxiety did not significantly increase. Working long hours was associated with higher odds, while teamwork and feeling appreciated at work were associated with lower odds, of stress, anxiety, and job burnout.

**Conclusions:**

Perceived stress and job burnout showed a mild increase over six months, even after exiting the lockdown. Teamwork and feeling appreciated at work were protective and are targets for developing organizational interventions to mitigate expected poor outcomes among frontline HCWs.

## Introduction

The novel coronavirus disease (COVID-19) pandemic has caused a devastating global health crisis in 2020 that has seen more than 100 million infected and 2 million deaths worldwide [1]. In some countries, maximum-capacity ICU admissions and mounting death tolls have significantly stressed healthcare systems, which are sustained by healthcare workers (HCWs) working tirelessly on the frontlines. The long-term stress, anxiety and job burnout experienced by HCWs are thus important to investigate.

**Fig 1:**
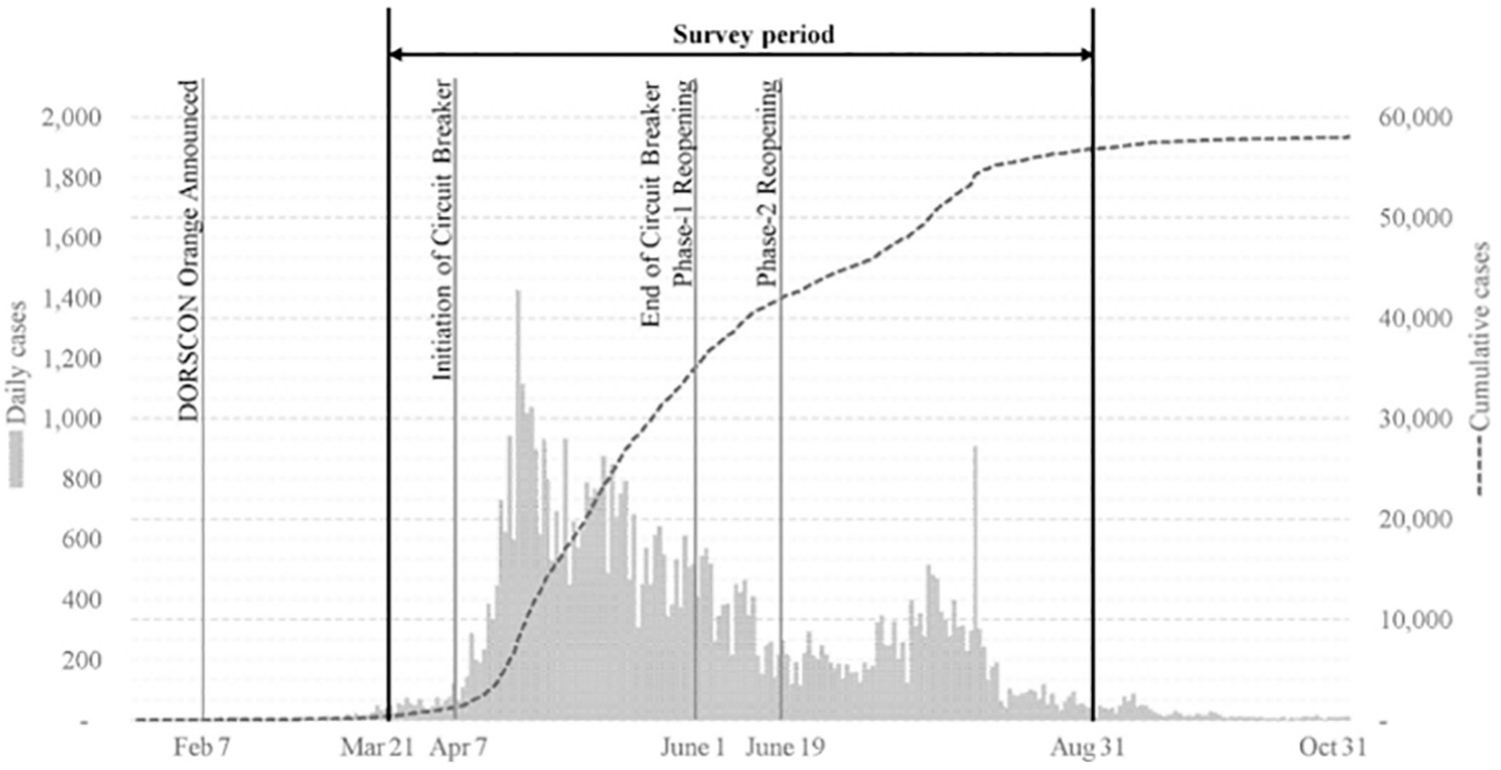
Timeline of COVID-19 cases in Singapore and study survey period.

Singapore is a densely populated city state of 5·69 million residents. Between the six-month period of March 1 to August 31, 2020, confirmed cases of COVID-19 rose from 106 to 56,812 (Fig 1). The sharp rise in cases over the April-June period was largely attributed to several outbreak clusters in migrant worker dormitories, with 95% of the nation’s cases coming from these clusters [2]. A nationwide lockdown (called Circuit Breaker) was instituted between April 7-June 1 [3]. Although Singapore’s ICU capacity has not been compromised and the overall death rate of 0·05% is considered low [4], the country has been at the second highest alert level of the national 4-level Disease Outbreak Response System Condition since February 2020, and healthcare systems have introduced many new protocols to increase vigilance and precautionary measures [5].

It is posited that being in such a state of stress and vigilance for a prolonged period of time will lead to negative psychological outcomes for HCWs. Early studies have reported elevated rates of chronic stress, anxiety states and job burnout among HCWs [6–8]. Reports of psychological morbidities appear to be widespread among frontline health workers, with a meta-analytic study suggesting that 41% of HCWs reported psychological distress during the COVID-19 period [9]. These results are unsurprising given the significant stressors facing frontline workers during the pandemic (e.g., personal safety and that of family and colleagues, long work hours, shortage of protective equipment) and highlight the importance of research focusing on those working on the frontlines. However, existing studies have mostly been cross-sectional in design and examined HCW outcomes only at specific time points in the pandemic.

The current study prospectively examined changes in the proportion of HCWs reporting stress, anxiety, and job burnout over a period of six months encompassing the peak of the outbreak and lockdown in Singapore. We hypothesized that the rates of perceived stress, anxiety, and job burnout among HCWs would increase over time. We were also interested to know the extent *objective job characteristics* (occupation, managerial role, experience of severe acute respiratory syndrome [SARS] as a HCW, years of working experience, level of exposure to COVID-19, working nightshifts, long work hours), *HCW-perceived job factors* (perceived job risk, teamwork, COVID-19-related communication, job dedication, feeling appreciated at work), and *personal resources* (self-efficacy, emotional support) were associated with the psychological outcomes of interest controlling for demographic, self-reported health, and time factors. The Job Demands-Resources Model [10, 11] of employee well-being and suggests that job demands (e.g., burnout) may be offset by resources, which may be provided/cultivated at work (e.g., teamwork, positive work environment) or be intrinsic to the individual (e.g., self-efficacy). We hypothesized that HCW-perceived job factors and personal resources, which are malleable and may be targets of intervention, will be significantly associated with stress, anxiety, and job burnout. Understanding the risk or protective effect these factors have on the psychological outcomes of HCWs may be useful for developing targeted interventions or mitigating expected poor outcomes as the global pandemic continues.

## Materials and methods

### Study design

This study used the convenience sampling method to prospectively follow HCWs from four tertiary hospitals in Singapore that provided care to COVID-19 patients during the pandemic. Recruitment occurred throughout the duration of the study. The self-reported data were collected from 12 March—31 August 2020, which included the peak of the pandemic that year and the nationwide lockdown period that occurred between April 7-June 1, 2020.

### Participants & data collection

Doctors, nurses, allied health professionals, administrative and operations staff from several institutions (Singapore General Hospital, KKH Women’s & Children’s Hospital, Changi General Hospital, Sengkang General Hospital) within the largest public healthcare group in Singapore were invited through work email and/or staff portals to participate. There were no exclusion criteria. Participation was voluntary and those who were interested accessed the study through a web link or QR code. Participants provided their consent online before completing the initial survey and monthly follow-ups which took 15 and 10 minutes to complete, respectively. Follow-up survey links were sent directly to participants’ email address, which served as a way to link the responses over time. No other personal identifying information was collected. Research assistants who managed the data collection on the Qualtrics platform were not involved in data analyses. The survey was administered in English. The study was approved by the National University of Singapore IRB (S-20-081) and exempted from review by the SingHealth Centralized IRB (2020/2160).

### Measurements of study variables

#### Study outcomes

Stress was measured using the 4-item Perceived Stress Scale (PSS-4) [12]. A summed score ranging from 0–16 was calculated, and a median score threshold of ≥ 8 was used to indicate stress. Anxiety was measured using the 7-item Generalized Anxiety Disorder (GAD-7) scale [13]. A summed score ranging from 0–21 was calculated and the recommended threshold score of ≥ 10 was used. Job burnout was measured using a one-item burnout question from the Physician Work Life Scale where a score ≥ 3 indicated presence of job exhaustion [14]. The measure has been used with a range of HCWs including doctors, nurses, and administrative personnel [14]. Higher scores on all study outcomes indicated greater severity.

#### Objective job characteristics

Participants reported their number of working years in healthcare. The responses to the following questions were coded “yes”/ “no”: whether they had a supervisory role, experienced the 2003 SARS outbreak as a HCW, worked night-shifts in the past month and worked longer than usual hours in the past month. Exposure to COVID-19 was assessed by “How often does your job require you to come in contact with suspected/ confirmed COVID-19 patients/ specimens?” with the response options being “not at all”, “occasionally”, and “daily”.

#### HCW-perceived job factors

Perceived job risk was assessed using the item “I feel that my job puts me at great risk of exposure to COVID-19” where responses ranged from “strongly agree” to “strongly disagree” on a 6-point scale which was later recoded into a binary variable (high risk vs. low risk) [15]. Effective COVID-19 communication in the workplace was assessed using three items: availability/ timeliness of updates, trustworthiness of information, and clarity of policies and protocols. Teamwork was assessed via the statement “My work team has been working well together”. The response options were “yes”, “neutral”, and “no”. These workplace support questions were considered to have face validity and adapted from a previous SARS outbreak study [16]. Job dedication was measured using the subscale from the Utrecht Work Engagement Scale-9 [17], where a higher summed score indicated higher job dedication, which consisted of feelings of enthusiasm, inspiration and pride for one’s job. Feeling appreciated was assessed by the statement “I feel appreciated by my department/ hospital/ employer” where the responses were coded into a binary variable: “never”/ “rarely” vs. “sometimes”/ “always”.

#### Personal resources

The 4-item short forms of the Patient-Reported Outcomes Measurement Information System (PROMIS) [18] measures of emotional support [19] and general self-efficacy [20] were used. The Emotional Support scale assesses perceived feelings of being cared for and valued as a person, while the General Self-efficacy scale measures confidence in exerting control over one’s situation. Both measures were rated to on a 5-point scale, with higher scores indicating increase in the construct measured. Summed scores were converted into T-scores.

#### Self-reported health

Presence of a chronic health condition was assessed by the question “In your lifetime, have you ever been diagnosed by a physician as having a chronic disease or medical condition?” with the response options “yes”, “no” and “neutral”. Whether a participant had been quarantined during the duration of the study was coded as “yes”/ “no”.

### Data analysis

A random-intercept logistic regression model, which is robust when missingness depends only on observed data (i.e., missing at random [MAR]), was used to investigate two questions: (1) whether rates of stress, anxiety, and job burnout among HCWs were increasing over time, and (2) what were the predictors of stress, anxiety, and job burnout among HCWs. To address the first question, we regressed the psychological outcomes of interest on calendar month. Calendar months were initially specified as a categorical variable to visualize trends, and later as a continuous variable to test for any statistically significant linear or quadratic trends.

To address the second question, we regressed the psychological outcomes of interest on potential predictors, while controlling for calendar month, demographic factors (age, gender, marital status, presence of a chronic health condition and living with children, elderly, or vulnerable persons), and placement on quarantine related to COVID-19. Predictors considered include objective job characteristics, HCW-perceived job factors and personal resources which were described earlier. Regression models omitting HCW-perceived job factors and personal resources were also estimated to investigate how predictive objective job characteristics are of the outcomes in the absence of information on subjective factors. Analyses were conducted using Stata version 15·1 [21] and statistical inference was based on cluster-robust standard errors (*SE*s) at the individual level [22] and the 5% significance level.

## Results

A total of 2744 HCWs participated in the survey. The study had a rolling admission and proportions of the sample completing their initial survey were 34% in March, 38% in April, 24% in May, 3% in June and 1% in July. Participants responded an average of two surveys. The majority of the sample were nurses (60%), female (81%), and lived with others considered vulnerable (children, elderly, or immunocompromised individuals; 57%). A small proportion reported being put on quarantine due to COVID-19 at some point during the study period (9%). The HCWs in this sample reported daily, occasional, and no contact with suspected/ confirmed COVID-19 cases at rates of 20%, 48% and 33% respectively. Refer to Table 1 for breakdown by occupation and further details.

**Table 1:** Healthcare worker characteristics (*N* = 2744).

	All	Doctors	Nurses	Allied health professionals	Others
	(*N* = 2744)	(*n* = 383)	(*n* = 1637)	(*n* = 409)	(*n* = 315)
**Mean (SD) or Frequency (%)**					
**Demographics**					
Female	2227	201	1473	316	237
	(81%)	(52%)	(90%)	(77%)	(75%)
Male	517	182	164	93	78
	(19%)	(48%)	(10%)	(23%)	(25%)
Age	38·86	37·80	34·75	35·36	39·95
	(10·59)	(9·95)	(10·31)	(10·10)	(11·99)
Marital status					
Single	1214	152	729	217	116
	(44%)	(40%)	(45%)	(53%)	(37%)
Married	1445	223	857	184	181
	(53%)	(58%)	(52%)	(45%)	(58%)
Divorced/Separated	68	8	43	5	12
	(3%)	(2%)	(3%)	(1%)	(4%)
Widowed	17	0	8	3	6
	(1%)	(0%)	(1%)	(1%)	(2%)
**Self-reported Health**					
Living with vulnerable household members	1558	225	890	251	192
	(57%)	(59%)	(54%)	(61%)	(61%)
Have chronic medical condition	567	73	331	80	83
	(21%)	(19%)	(20%)	(20%)	(26%)
Been on quarantine due to COVID-19	233	30	133	53	17
	(9%)	(8%)	(9%)	(14%)	(6%)
**Job Characteristics**					
Work experience as HCW					
<5 years	624	81	314	119	110
	(23%)	(21%)	(19%)	(29%)	(35%)
5–9 years	725	89	449	102	85
	(27%)	(23%)	(28%)	(25%)	(27%)
10–14 years	651	75	439	89	48
	(24%)	(20%)	(27%)	(22%)	(15%)
15+ years	727	137	428	94	68
	(27%)	(36%)	(26%)	(23%)	(22%)
Managerial/Supervisory role	771	194	344	155	78
	(28%)	(51%)	(21%)	(38%)	(25%)
Experienced SARS as HCW	589	91	358	75	65
	(22%)	(24%)	(22%)	(18%)	(21%)
Contact with COVID-19 cases					
No contact	900	67	366	240	227
	(33%)	(18%)	(22%)	(59%)	(72%)
Occasional contact	1306	254	866	124	62
	(48%)	(66%)	(53%)	(30%)	(20%)
Daily contact	537	62	404	45	26
	(20%)	(16%)	(25%)	(11%)	(8%)
Long work hours	1045	144	675	126	100
	(40%)	(40%)	(43%)	(33%)	(34%)
Worked night shifts	1394	183	1107	65	39
	(53%)	(50%)	(71%)	(17%)	(13%)

*Note*. Frequencies may not add up to total sample size due to missing responses.

The majority (71%) of HCWs perceived their job to put them at high risk of exposure to COVID-19. The majority perceived COVID-19-related communication to be timely (82%), trustworthy (82%) and clear (63%); that their team worked well together (76%); and that they felt appreciated at work (80%). Elevated perceived stress, anxiety, and job burnout were reported by 33%, 13% and 24% of the overall sample at baseline, respectively; nurses reported the highest rates across all study outcomes. Refer to Table 2 for further details.

**Table 2:** Healthcare worker-perceived job factors, personal resources, and study outcomes at initial survey (*N* = 2744).

	All	Doctor	Nurse	Allied health	Others
	(n = 2744)	(n = 383)	(n = 1637)	(n = 409)	(n = 315)
**Mean (SD) or Frequency (%)**					
**Perceived job factors**					
Job risk					
High	1882	263	1264	208	147
	(71%)	(71%)	(80%)	(53%)	(49%)
Low	758	106	318	180	154
	(29%)	(29%)	(20%)	(46%)	(51%)
Effective COVID-19 communication					
Available/timely updates (Yes)	2100	296	1253	313	238
	(82%)	(82%)	(81%)	(83%)	(82%)
Trustworthy information (Yes)	2110	317	1242	319	232
	(82%)	(88%)	(80%)	(85%)	(80%)
Clear protocols and policies (Yes)	1617	197	977	239	204
	(63%)	(55%)	(63%)	(64%)	(70%)
Teamwork					
Yes	1948	311	1137	283	217
	(76%)	(86%)	(73%)	(76%)	(75%)
Neutral	580	42	393	80	65
	(23%)	(12%)	(25%)	(21%)	(22%)
No	33	4	15	10	4
	(1%)	(1%)	(1%)	(3%)	(1%)
Not applicable	17	4	6	2	5
	(1%)	(1%)	(0·5%)	(1%)	(2%)
Feel appreciated at work					
Never/Rarely	507	60	303	80	64
	(20%)	(17%)	(20%)	(21%)	(22%)
Sometimes/Always	2071	301	1248	295	227
	(80%)	83%)	(80%)	(79%)	(78%)
Job dedication	12·52	13·52	12·36	12·42	12·33
	(3·35)	(3·07)	(3·39)	(3·12)	(3·54)
**Personal resources**					
Self-efficacy	44·87	46·96	44·56	44·30	44·64
	(7·90)	(7·63)	(7·77)	(8·00)	(8·40)
Emotional Support	50·40	52·08	50·22	50·45	49·21
	(8·46)	(7·63)	(8·55)	(8·42)	(8·74)
**Study outcomes**					
Stress (PSS-4)	6·10 (2·61)	5·74 (2·50)	6·21 (2·64)	6·05 (2·49)	5·98 (2·66)
score ≥ 8	33%	26%	36%	29%	28%
Anxiety (GAD-7)	4·96 (4·47)	4·58 (4·20)	5·07 (4·58)	4·94 (4·12)	4·91 (4·59)
score ≥10	13%	11%	14%	12%	13%
Job burnout (PWLS-1)	2·12 (0·79)	1·99 (0·70)	2·18 (0·81)	2·13 (0·73)	1·95 (0·77)
score ≥3	24%	17%	27%	22%	16%

*Note*. Frequencies may not add up to total sample size due to missing responses.

### Change over time

**Fig 2:**
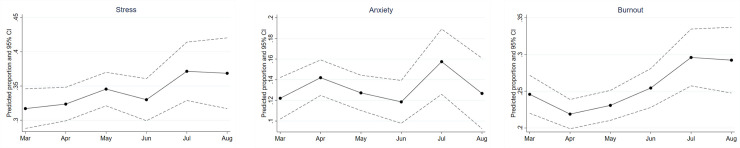
(a)-(c). Proportion of healthcare workers reporting stress, anxiety and job burnout. (a). Proportion of healthcare workers reporting stress between March-August 2021. (b). Proportion of healthcare workers reporting anxiety between March-August 2021. (c). Proportion of healthcare workers reporting job burnout between March-August 2021. *Note*. Predicted proportions estimated from a random intercept model without additional controls.

Fig 2 plots predicted the proportion of HCWs reporting stress, anxiety, and job burnout by calendar months, accounting only for random individual effects. Fig 2(a) and 2(c) show that the proportion of HCWs reporting stress and job burnout were trending upwards over the period of March to August. In particular, there was an increase in proportion of HCWs reporting job burnout in July and August relative to April, which was when the lockdown was implemented and when hospital services were cut back (*p* = ·001). While the proportion reporting anxiety did not exhibit a clear increasing trend, the timing of its fluctuations upwards appeared to coincide with the implementation of the lockdown in April/ May and resumption of full hospital services in July.

Specifying calendar months as a continuous variable, we used the same regression model (controlling only for random individual effects) to test for linear and quadratic trends. We found statistically significant positive linear trends for stress (*OR* = 1·08, *SE* = 0·04, *p* < ·05) and job burnout (*OR* = 1·16, *SE* = 0·05, *p* < ·01), implying that the proportion of HCWs who reported being stressed and burnt out increased by approximately 1·0% and 1·2% respectively per month over the study period. The quadratic term for calendar months was significant only for job burnout (*p* < ·05).

### Predictors of stress, anxiety, and job burnout

For each outcome, we estimated two models (Table 3). Model 1 examined the association between outcomes of interest and objective job characteristics only while controlling for demographic, self-reported health, and time factors. Working long hours was significantly associated with stress, anxiety, and job burnout. Occupation was also related to the study outcomes. Compared to doctors, nurses were more likely to report higher stress, AHPs more likely to report job burnout, and other HCWs were more likely to report anxiety. Compared to HCWs with the least work experience, HCWs worked 5–9 years were more likely to report job burnout. Being in a managerial/supervisory role was associated with lower stress.

**Table 3:** Predictors of stress, anxiety, and job burnout.

	Model 1			Model 2		
	Stress	Anxiety	Job Burnout	Stress	Anxiety	Job Burnout
***Objective Job Characteristics*:**						
Doctor	Ref.	Ref.	Ref.	Ref.	Ref.	Ref.
Nurse	1·79**	1·43	1·63^†^	1·11	0·88	1·02
	(0·36)	(0·43)	(0·43)	(0·21)	(0·26)	(0·26)
Allied Health	1·25	1·39	1·85*	0·82	0·91	1·35
	(0·31)	(0·49)	(0·58)	(0·19)	(0·32)	(0·40)
Others	1·22	2·21*	1·17	0·71	1·29	0·75
	(0·34)	(0·86)	(0·42)	(0·19)	(0·51)	(0·26)
Managerial/Supervisory role	0·72*	0·76	1·01	0·88	0·99	1·24
	(0·12)	(0·17)	(0·21)	(0·13)	(0·23)	(0·24)
Experienced SARS as HCW	0·68	1·22	0·83	0·73	1·40	0·98
	(0·18)	(0·48)	(0·30)	(0·18)	(0·53)	(0·33)
< 5 years’ experience	Ref.	Ref.	Ref.	Ref.	Ref.	Ref.
5–9 years’ experience	1·12	0·97	1·72*	0·97	0·78	1·44^†^
	(0·21)	(0·25)	(0·40)	(0·18)	(0·21)	(0·31)
10–14 years’ experience	0·87	0·73	1·33	0·83	0·67	1·37
	(0·21)	(0·25)	(0·39)	(0·19)	(0·23)	(0·38)
15+ years’ experience	1·18	1·17	1·14	1·03	0·10	1·19
	(0·37)	(0·55)	(0·46)	(0·31)	(0·47)	(0·45)
No COVID-19 contact	Ref.	Ref.	Ref.	Ref.	Ref.	Ref.
Occasional COVID-19 contact	1·19	1·04	1·37^†^	1·06	0·89	1·41*
	(0·17)	(0·20)	(0·23)	(0·14)	(0·18)	(0·23)
Daily COVID-19 contact	1·25	1·28	1·11	1·37^†^	1·19	1·46^†^
	(0·22)	(0·31)	(0·24)	(0·23)	(0·29)	(0·30)
Work night shift	0·95	1·11	1·30	1·06	1·25	1·48*
	(0·13)	(0·22)	(0·23)	(0·15)	(0·25)	(0·26)
Long work hours	1·95**	2·85**	4·12**	1·63**	2·27**	3·36**
	(0·22)	(0·46)	(0·58)	(0·18)	(0·38)	(0·46)
***HCW-Perceived Job Factors*:**						
Team work well together	-	-	-	0·59**	0·69*	0·55**
				(0·08)	(0·13)	(0·08)
Effective COVID-19 communication	-	-	-	0·90* (0·05)	0·91 (0·07)	0·94 (0·06)
Job dedication	-	-	-	0·87**	0·85**	0·76**
				(0·02)	(0·03)	(0·02)
Perceived job risk	-	-	-	1·29**	1·80**	1·11
				(0·11)	(0·24)	(0·12)
Feel appreciated at work (Sometimes/Always)	-	-	-	0·65** (0·09)	0·41** (0·08)	0·34** (0·05)
***Personal Resources*:**						
Emotional support	-	-	-	0·95**	0·97**	0·98*
				(0·01)	(0·01)	(0·01)
Self-efficacy	-	-	-	0·92**	0·94**	0·96**
				(0·01)	(0·01)	(0·01)
Observations	4857	4857	4858	4758	4758	4759

SARS = Severe Acute Respiratory Syndrome; HCW = healthcare worker

Coefficients represent odds ratios. Cluster-robust standard errors at the individual level are reported in parentheses. Additional controls not reported in the table include calendar month dummies, age, gender, marital status, presence of chronic health condition and living with children, elderly, or vulnerable persons), and placement on quarantine related to COVID-19. Statistical significance denoted by ^†^ p<0·10 * p<0·05, ** p<0·01.

Model 2 examined the contribution of HCW-perceived job factors and personal resources to the earlier model. Perceived high job risk was associated with increased stress and anxiety. Effective COVID-19 communication was associated with lower stress. As expected, perceived teamwork, job dedication, feeling appreciated at work, self-efficacy and emotional support were significantly and negatively associated with lower stress, anxiety, and job burnout. Most objective job characteristics that were predictive of psychological outcomes in Model 1 (i.e., occupation, years of working experience, and holding a managerial role) were no longer statistically significant with the inclusion of subjective factors (i.e., HCW-perceived job factors and personal resources) in Model 2. Only long work hours, working night shifts and frequency of COVID-19 contact remained significant in Model 2.

## Discussion

Our data show changes in the proportion of HCWs who reported stress, anxiety, and job burnout at the peak of the COVID-19 pandemic in Singapore. Prospective data collection was made possible by online, electronic surveys that allowed fast, efficient data collection with wide reach. The proportion of HCWs reporting stress and job burnout rose an average of 1·0% and 1·2% per month respectively over the study period, with indications of a U-shaped trend for rates of job burnout. These findings are consistent with our hypotheses and corroborate reports in the news worldwide that HCWs are feeling the brunt of the pandemic as it wears on. The generally mild increase over time found in the study may reflect the relative stability we see in Singapore, and the healthcare system’s ability to effectively respond to the outbreak, i.e., low community transmissions, wide-spread testing efforts, minimal COVID-19 transmissions to healthcare workers within the work setting, etc.

We found fluctuations in the proportion of HCWs reporting anxiety that potentially reflect pandemic-response events such as entering and exiting the lockdown. Given that anxiety may be viewed as a sequelae of maladaptive coping to stress, one would expect stress and anxiety to have similar trajectories. Instead, the proportion of HCWs reporting anxiety did not continue to increase after the lockdown (in contrast to stress), suggesting that HCWs are resilient and had ways to cope that allowed their anxiety to return to earlier levels after pandemic-response events.

The trajectory of job burnout is one that appears to reflect how busy workload was for the majority of HCWs during the study period. We see that rate of job burnout decreased during the lockdown when many elective services/ procedures in the hospital were temporarily halted and split-team arrangements were implemented, which then progressively increased as hospitals resumed services, dealt with the backlog of cases, and had an increased number of patients who were admitted for suspected COVID-19 (e.g., patients with community-acquired pneumonia). Additionally, HCWs had to adjust to newly-implemented infection control protocols and audits, while dealing with patients and families that were also adjusting to new hospital policies such as mandatory swabs, restricted visitations, etc. The rates of job burnout in July and August were significantly higher than March pre-lockdown, however it is unclear whether they are significantly different than pre-COVID-19 rates as we do not have the data for comparison. However, our early rate of job burnout (ie, 25% in March) corresponds to that of palliative care HCWs in Singapore pre-COVID-19 that indicated 26% of their respondents reported emotional exhaustion (an aspect of burnout which is similar to what we measured) [23].

HCW-perceived job factors and personal resources, as compared to objective job characteristics, were found to be significantly associated with stress, anxiety, and job burnout. Although the frequency of COVID-19 contact and night shifts were significantly associated with job burnout, only reports of working longer hours than usual appeared to be strongly and consistently associated with stress, anxiety, and job burnout. The effects of long work hours is compounded by not having time-off to rest and recharge. Anecdotal reports indicate HCWs taking minimal time off in the last year, partly due to increased workload post-lockdown and partly due to international travel restrictions. Future studies may want to consider residential status of healthcare workers, especially when a substantial proportion of the healthcare workforce consists of HCWs who are non-residents or with families overseas.

Previously published studies on HCW well-being during the COVID-19 pandemic have reported various job risk factors, notably that nurses and those with a higher degree of exposure to suspected/ confirmed cases have poorer outcomes [7, 8, 24]. Our data indicate that there are factors beyond objective job characteristics that are important to consider, e.g., perceiving one’s job to be high risk increased the odds of stress and anxiety by 29% and 80% respectively. Our findings are consistent with prior studies examining the role of perceived risk [25, 26] and highlight the opportunities leaders in positions of authority have to shape and influence these perceptions of risks, especially in the context where there may be fear among HCWs due to misinformation, e.g. over-inflation of the transmissibility of the virus or its sequelae.

A number of protective factors emerged, with the two most important ones being teamwork and feeling appreciated at work. HCWs who reported that their team(s) worked well together and that they felt appreciated at work sometimes or always had 31–45% and 35–66% respectively lower odds of being stressed, anxious, and burnt out. Being able to trust and depend on one another and feeling valued are intuitively important in times like this, and our data substantiates this. There have been countless efforts around the world to show appreciation to various groups of frontline workers throughout the pandemic, and indeed, they are important morale boosters. A less well-publicized strategy relates to efforts to improve or maintain camaraderie and teamwork among HCWs. This may be particularly important for those on the frontlines who are exhausted, crave human connection, yet have been told to minimize contact with one another for safety purposes. Readers who are further interested about psychological principles that can improve teamwork in medical settings during crisis are referred to Traylor et al.’s review [27]. Consistent with findings in prior literature, other protective factors that were associated with mitigation of stress, anxiety, and job burnout include job dedication (i.e., feelings of enthusiasm, inspiration and pride for one’s job), emotional support (i.e., having others to confide in), and self-efficacy (i.e., feeling confident in one’s ability to cope) [28–30]. These resources are potentially important to build and nurture at the workplace, and will help the healthcare workforce be psychologically resilient during challenging times.

### Study limitations

We did not measure previous mental health history; this was a conscious decision made to respect HCW privacy. We did not have pre-COVID-19 data to compare prior rates of stress, anxiety, and job burnout; thus, our interpretation of the data is only within the study time frame. We had considerable dropouts where approximately half of our sample contributed one data point; this was dealt by using a random-intercept model. Our model assumes that data missingness depends only on observed data (ie, MAR); in the worst case scenario, we are underestimating rates of stress, anxiety, and job burnout as those who dropped out had higher odds of being stressed at their initial survey.

## Conclusions

Our prospective investigation found that during the six-month peak of the pandemic in Singapore, stress and job burnout increased mildly over time, while anxiety fluctuated according to pandemic-response events. We found that there are a number of protective work-related and personal resources that may be modifiable through initiatives and interventions to ameliorate HCW stress, anxiety, and job burnout, two of which are teamwork and feeling appreciated at work. Every country or hospital setting has its own unique features and challenges, and our hope is that the findings presented can be helpful in any way to others as we continue the ongoing worldwide battle.
